# Relationships between Body Size and Parasitic Fitness and Offspring Performance of *Sclerodermus pupariae* Yang *et* Yao (Hymenoptera: Bethylidae)

**DOI:** 10.1371/journal.pone.0156831

**Published:** 2016-07-01

**Authors:** Shangkun Gao, Yanlong Tang, Ke Wei, Xiaoyi Wang, Zhongqi Yang, Yanlong Zhang

**Affiliations:** Key Laboratory of Forest Protection, China State Forestry Administration; Research Institute of Forest Ecology, Environment and Protection, Chinese Academy of Forestry, Beijing, China; Federal University of Viçosa, BRAZIL

## Abstract

The relationship between body size and fitness in parasitoid wasps has several effects on parasitic ability, reproductive behavior in female wasps, and progeny fitness. Female wasps with various body sizes were obtained by mass–rearing a gregarious ectoparasitoid, *Sclerodermus pupariae*, which is one of the excellent parasites to control the larvae and pupae of Buprestidae and Cerambycidae. We investigated the effects of body size of adult females introduced on *Thyestilla gebleri* (Coleoptera: Cerambycidae) larvae on their paralysis time, pre–oviposition period, oviposition period and fecundity, and the related fitness of their offspring. Results showed that small female wasps needed more time to paralyze a host and had a higher mortality rate than large female wasps. More offspring were produced by large female wasps than by small female wasps, and the percentage and body size of female offspring was not affected by maternal body size. The duration of the egg stage was not affected by foundress size, nor was that of the pupal stage, but the duration of the larval stage and generation time of small female wasps was longer than that of large females. Our findings suggest that the parasitic fitness and offspring performance are affected by maternal size, and there is need to choose reasonable body size of female wasps, to optimally utilize mass rearing and to control target pests with the lowest mortality cost.

## Introduction

Body size is one of main determinants of history strategies in various animals, such as trophic niche parttioning in the rhinolophid bat species [1], life–history trade–offs in a marine fish [2], predatory strategies of spiders [3], the distrbution of caddisworms[4], warm–up rates and body temperatures in bees [5].

In insects, body size is also found to be positively correlated with fitness of their life–history traits [6]. Especially in hymenopteran parasitoids, “adult size–fitness hypothesis” shows that large size individuals confers more physiological and behavioral advantages, such as the ability to search and subdue large, high quality hosts, lifetime fecundity, longevity and mating success, even the outcome of numerous parasitoid–host interactions, than small size congeners [7–11]. Body size is often considered as one of the most prominent fitness functions for examining resource allocation and predicting clutch size and progeny sex allocation [11–13]. The optimal phenotype in most organisms is determined by a trade–off in life–history traits [14]. As one of phenotypes exhibited in parasitoids, body size maybe based on a trade–off between self–development and functional constraints.

Parasitoids were classified into two categories depending on attacking strategy: koinobiosis and idiobiosis. Koinobionts allow the host to continue developing and larvae usually feed within the host body, whereas idiobionts stop host development and larvae usually feed externally. Idiobionts generally produce small numbers of large anhydropic eggs, whereas koinobionts generally produce copious numbers of small. Idiobionts usually enjoy longer lifespans than koinobionts [15, 16], but lower daily rates of oviposition [17]. Because ecophysiological selection pressures are different, offspring sizes of idiobionts are usually correlated with parasitized host sizes and development times are often uniform [18–21], except for these fitness parameters of some idiobiont parasitoids vary with host size and host species [22], whereas cases with koinobionts are much more complicated [19, 23, 24].

The gregarious idiobiont ectoparasitoid *Sclerodermus pupariae* Yang *et* Yao (Hymenoptera: Bethylidae) is a recently discovered species, found in the pupa and later larva of the emerald ash borer *Agrilus planipennis* Fairmaire (Coleoptera: Buprestidae) in Tianjin, China [25]. This wasp has up to five generations per year and has excellent host–searching and host–attacking abilities, and a high proportion of female offspring. Its natural parasitic rate is as high as about 10% and it produces an average of 35 offspring (one to four males) in a single larva of *A*. *planipennis*. As it can tolerate lower temperatures, it has a potentially more extensive range of application than *S*. *guani* Xiao *et* Wu and *S*. *sichuanensis* Xiao, which have been widely used to control wood–boring insect pests [26]. Currently, *S*. *pupariae* has been used for the natural biological control of several buprestid and cerambycid pests [27–29]. The female parasitoid stabbed toxins to paralyze the searched host larva with its acicular ovipositor. Because *S*. *pupariae* is a synovigenic parasitoid, then it started to clean the paralyzed host and feeded on hemlymph outflow by biting into the epidermis before oogenesis. Throughout the whole developmental duration of offspring, the mother parasitoid visited her offspring via touching her offspring with mouthparts and patting them continuously with antennae and provided care, including replacement of eggs fell off from host surface, dispersion of overlapped larvae, remove of dead or melanotic offspring far from the host and biting a hole in the cocoon for the emerging progeny [25, 27]. Adaptive learning significantly enhances the ability of *S*. *pupariae* to utilize target host larvae [30]. Except for target pests, other wood pests (buprestid and cerambycid larvae) can be used as alternative hosts for *S*. *pupariae* to maintain their population and thus sustain pest control in the field [28, 29]. *S*. *pupariae* can optimize its developmental times and progeny size based on the abundance of different–sized host beetle larvae [31].

With the wide application of biological control technologies, natural enemy insects mass reared on substitute hosts are an important part of biological control success [32]. In our laboratory, populations of *S*. *pupariae* have been bred using the substitute hosts *Thyestilla gebleri* Faldermann larvae for many years. The reason for using these long–horned beetle larvae is their suitable body size (0.1–0.4 mg) and weaker defense behaviors. The nutritions in a single overwintering *T*. *gebleri* larva meet a generation development of one female *S*. *pupariae*. Due to different rearing techniques, such as different female numbers introduced on one host, female wasps with obvious body size difference have been produced during the mass–rearing process. In previous studies, it has been found that many factors contributed to the generation of multiple body sizes of *Sclerodermus* parasitoid wasps, including maternal effects [33, 34], population depression [35], parasitoid–host ratio [36], maternal care [37], and the ability to compete for and successfully parasitize hosts [38]. All of the above factors, except for inheritance from mother wasps, they exert their effects indirectly through influencing host quality and nutrition.

However, little is still known about the relationship between development time on parasitoid fitness and adult size [24, 39]. Here, the aim of our study was to examine the effect of maternal body size on parasitic fitness and its offspring performance. We compared parasitism parameters of mothers (host paralysis time, pre–oviposition period, oviposition period, and fecundity), and performance parameters of their offspring (development durations, numbers of female offspring, sex ratio, and body sizes of female offspring).

## Materials and Methods

### Experimental materials

Laboratory populations of *S*. *pupariae* that parasitize *A*. *planipennis* larvae were established from individuals collected in the late autumn of 2011 at Guangang Forest Park, Dagang District in Tianjin, China. In the laboratory, the parasitoid was reared solely on larvae of *T*. *gebleri* for thirty generations prior to the experiment to exclude any possible host source effects. The different numbers of female parasitoids (one to eight) were presented with a larval of *T*. *gebleri*, then their female offspring with various body size were selected for the experiments. All the experimental female parasitoids were taken from bloods one week after emergence, thus ensuring they were mated. Each mated parasitoid was introduced into a glass vial (diameter 1 cm, length 5 cm), containing a host larva. The vial was blocked tightly with a cotton plug. Then, they were placed in plastic boxes (12 × 7 × 5 cm) and maintained at 25 ± 5°C, 60–70% relative humidity, under a light: dark regime of 10:14 h.

After introduction into the vial, some individuals immediately started stinging the host, whereas others moved over the cotton plug to escape. Females stabbed at the end of host’s abdomen with their acicular ovipositor, and secreted toxins to paralyze the host larvae. The host was considered paralyzed when it stopped struggling and the head capsules remained motionless. Then female wasps started to clean the paralyzed host and absorb hemolymph outflow via biting into the epidermis near the palate. Wasps gave priority to laying eggs on the inter–segmental membranes of the host, and continued laying eggs until the entire host was covered with eggs. During the developmental duration of offspring, the mothers provided care and did not leave until progeny emergence.

The *T*. *gebleri* larvae used as a substitute host in mass rearing *S*. *pupariae* were collected from Tianjin, China. It mainly attacks fiber crops and overwinters as larvae in the roots of hosts in Tianjin. The collected overwintering larvae were stored at 5°C to maintain them alive and fresh. Host larvae were weighted using an analytical balance (sensitivity 0.1mg), and a total of 111 larvae (200.0 – 240mg) were chosen and randomly separated into individual vials (diameter 1 cm, length 6 cm,). Although there was a 40 mg variation among the hosts used, our preliminary test (*R* Foundation for Statistical Computing) indicated that the relationships between host weight and parasitic parameters (host paralysis time, pre–oviposition period, oviposition period, fecundity, development durations of offspring, numbers of female offspring, sex ratio, and body sizes of female offspring) were not significant (i.e. paralysis time: *F* = 4.02, *df* = 1, 93, *P* = 0.417), and we were effectively treating host size as a constant.

All necessary permits were obtained for the described field studies. The Guangang Forest Park is managed directly by the government of Dagang District, Tianjin Municipality, China. The secretary of the department of forestation, Guangang Forest Park, named Gui-jun Liu have issued the permission for field studies. All the samples of this study did not involve endangered or protected species.

### Body size experiments

Each female wasp was led into a capillary glass tube (diameter 1 cm, length 5 cm,) and the body length measured with a micrometer mounted on a stereo-microscope (sensitivity of 0.001 mm). Body size was measured as the length from the head to the tip of the abdomen. A total of 111 mated females with a range of body size from 2.0 mm to 3.2 mm were chosen and separated into three classes based on length increments of approximately 0.40 mm. We used three groups: small (2.00–2.39 mm), medium (2.40–2.79 mm), large (2.80–3.2 mm) with 41, 47 and 23 individuals (replicates) for each group respectively. Each female was inoculated in a vial containing a single *T*. *gebleri* larva. The opening of each vial was blocked with a cotton plug. All vials were maintained in an artificial–climate chamber at the same conditions as the female wasps were bred (25 ± 5°C, 60–70% relative humidity, under a light: dark regime of 10:14 h).

Adult and offspring performance were recorded for 30–40 days after the female wasps were introduced. The paralysis time (days), pre–oviposition period (days), oviposition period (days), fecundity (number of offspring wasps that emerged) and time (days) offspring spent at the egg, larval, and pupal stages were observed and recorded under a stereo–microscope every 12 h. All parasitized hosts were monitored twice a day under a microscope. We recorded the period between inoculation and the first wasp sting (to the host) and the onset of paralysis in host larvae as host paralysis time. The pre–oviposition period of adult females was defined as the time from host paralysis to first reproduction. The oviposition period was defined as the time between the first and last eggs laid. The developmental duration of parasitoid offspring (oviposition to emergence) were recorded for each replicate. The duration of the egg stage was calculated as the time interval between the laying of the first egg laid and the first larval emergence. The duration of the larval stage was identified as the period between the emergence of the first larva and the first cocoon. The duration of the pupal stage was defined as the time interval between the emergence of the first cocoon and the first adult. The developmental duration of offspring was calculated as the time from eggs to adults. Generation time was calculated as the total time from the foundress’s sting to the emergence of her adult offspring. Other offspring performance characteristics were average body size of 15 emerged adults randomly selected from each group and the sex ratio (proportion of females). Mortality was assessed daily and deteriorated hosts were excluded.

### Data analysis and statistics

Statistical analyses were performed using GraphPad Prism version 5.0 for Windows (GraphPad Software, San Diego, CA). Kaplan–Meier survival curves between the cumulative survival percentages and adult parasitoid reproduction times (paralysis time, pre–oviposition period and oviposition period) were conducted and the log–rank tests were used to assess their differences in the three treatments (foundress body sizes). Analyses of variance (ANOVA), followed by Tukey’s separation of means test, were used to compare body size of foundresses, the developmental duration of offspring, generation time (male and female wasps) and sex ratio in three treatments. Chi–square test was used to compare the performance of female wasps when attacking host larvae. Regression analyses were used to describe the various relationships between fecundity (number of offspring emerged), numbers of female offspring, body size of female offspring and body size of foundresses arcsine square root transformation was applied to the percentage–based data (sex ratio), and the transformed data were used for analysis.

## Results

### Adult performance with various body sizes

The three groups of foundresses had significantly different body sizes (*F* = 298.3, *df* = 2, 108, *P* < 0.0001). Averages for each group were: 3.06 ± 0.18 (large), 2.59 ± 0.09 (medium), and 2.31 ± 0.10 (small).

**Fig 1:**
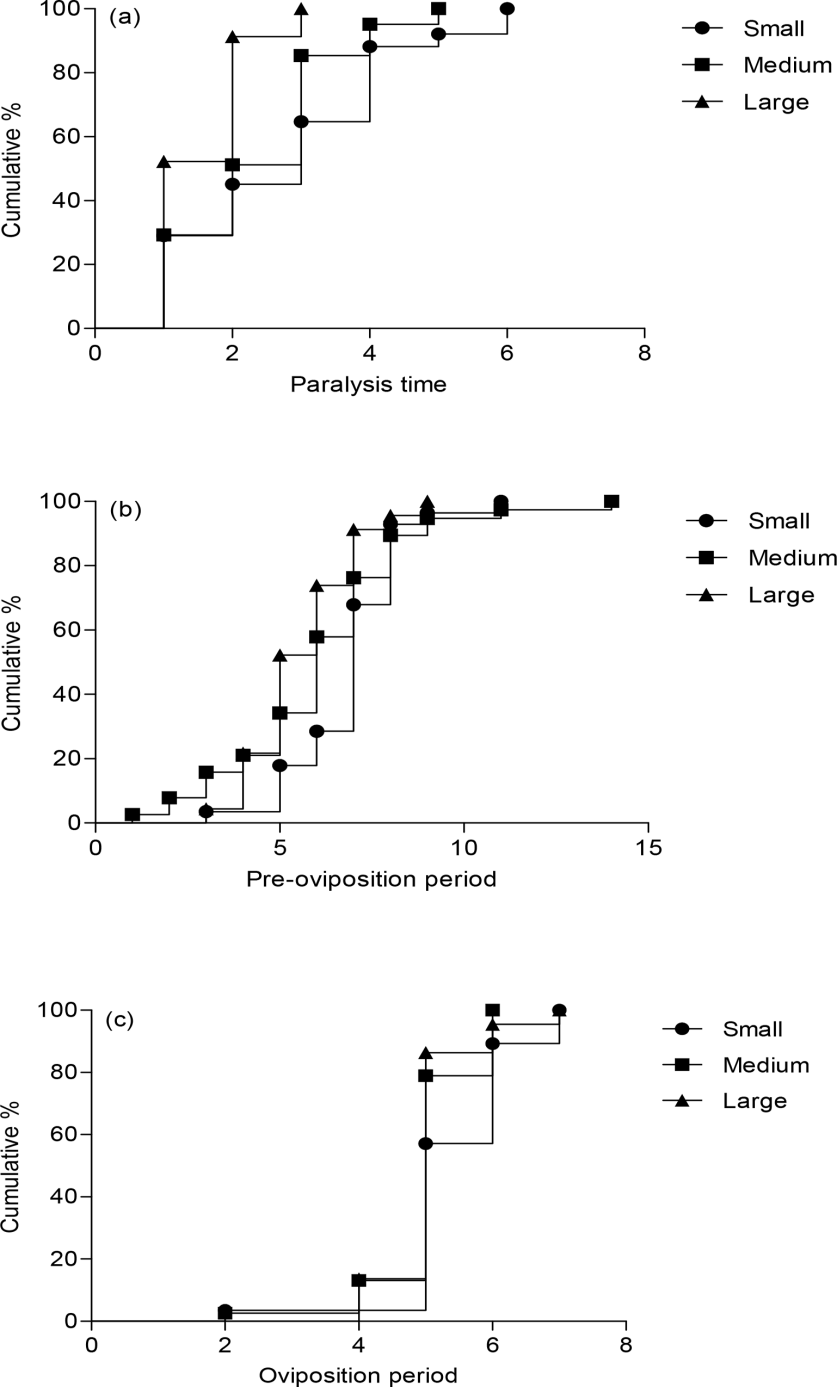
The reproduction time of *Sclerodermus pupariae* with various body sizes. (a) paralysis time (*Chi–square* = 9.424, *df* = 2, *P* = 0.009), (b) pre–oviposition period (*Chi–square* = 4.75, *df* = 2, *P* = 0.093), and (c) oviposition period (*Chi–square* = 2.258, *df* = 2, *P* = 0. 323) (Log–rank test).

Adult performance of female wasps varied significantly among the three size classes (Table 1), as indicated by mortality (*Chi–square* = 7.29, *df* = 2, *P* = 0.03). The cumulative percentages of paralyzed hosts as paralysis time progressed were determined (Fig 1A). Small female wasps took significantly more time to paralyze hosts (*Chi–square* = 9.42, *df* = 2, *P* < 0.05) and had much higher mortality than larger female wasps when attacking the host larvae (*Chi–square* = 7.29, *df* = 2, *P* = 0.03) (Table 1). However, once host larvae were paralyzed, all sizes of female wasps could oviposit (*Chi–square* = 0.55, *df* = 2, *P* = 0.76) (Table 1) and their offspring could develop to adults with no differences among large, medium, small treatments (*Chi–square* = 2.03, *df* = 2, *P* = 0.36) (Table 1). The pre–oviposition and oviposition periods had no significant difference among the three groups (Fig 1B, *Chi–square* = 4.75, *df* = 2, *P* = 0.09; Fig 1C, *Chi–square* = 2.26, *df* = 2, *P* = 0.32).

**Table 1:** Costs to females of *Sclerodermus pupariae* of attacking *Thyestilla gebleri* larvae.

	Number	Proportion	Proportion	Proportion
	Replicates	Wasps dead	Wasps that laid eggs	Wasps whose offspring emerged
**Small wasps**	41	10/41 a	28/31 a	26/28 a
**Medium wasps**	47	6/47 b	38/41 a	35/38 a
**Large wasps**	23	0/23 c	22/23 a	18/22 a
**Chi–square**		7.29	0.55	2.03
***P***		0.03	0.76	0.36

Different letters indicate significant differences at *P* < 0.05 (Chi–square test).

**Fig 2:**
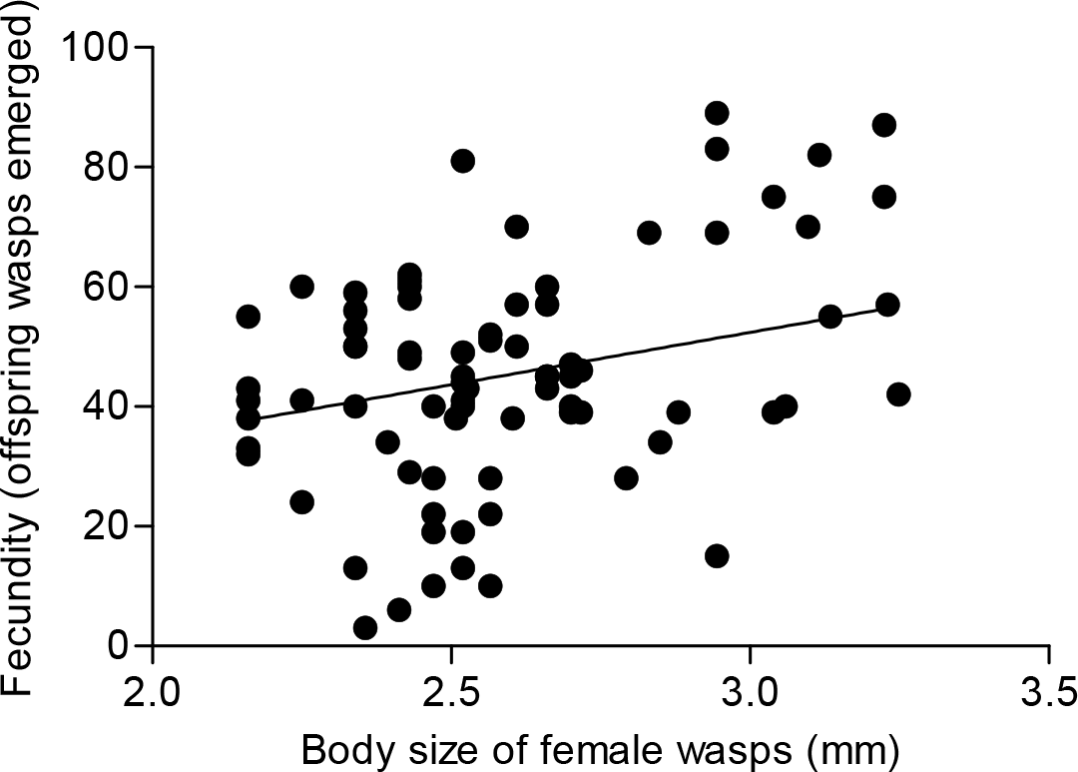
Relationship between body size of female wasps and fecundity (number of offspring emerged). Effect of body size on fecundity (number of offspring emerged) is significant (*y* = 25.434*x* – 21.136, *R*² = 0.1495, *F* = 13.53, *df* = 1, 77, *P* < 0.0001).

Furthermore, the fecundity (numbers of offspring emerged) of large female wasps was significantly higher than that of small female wasps (*F* = 6.38, *df* = 2, 76, *P* = 0.003). Results from regression analysis showed a significant linear relationship between fecundity and body size of female wasps (*F* = 13.53, *df* = 1, 77, *P* < 0.0001), the number of progeny significantly increased with mother body sizes (Fig 2).

### Offspring performance

**Fig 3:**
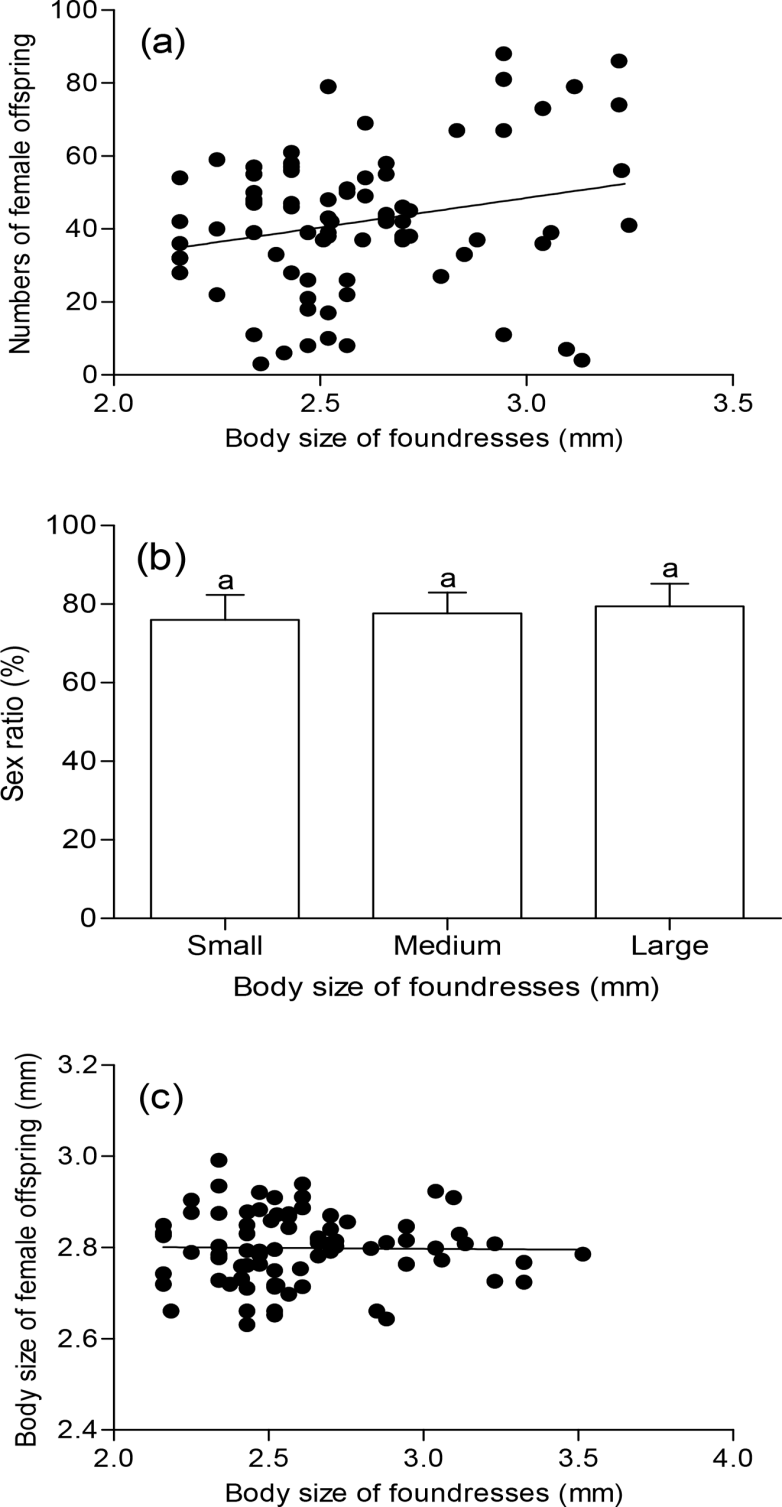
Offspring performance of *Sclerodermus pupariae* with mothers of various body sizes. (a) numbers of female offspring (*y* = 16.394*x* – 0.7234, *R*² = 0.0581, *F* = 4.75, *df* = 1, 76, *P* = 0.032), (b) sex ratio (proportion of females) (*F* = 2.03, *df* = 2, 75, *P* = 0.14), (c) body size of female offspring (*y* = –0.0024*x* + 2.8051, *R*² = 8 × 10^−5^, *F* = 0.006, *df* = 1, 76, *P* = 0.94). Bars indicate standard errors and different letters indicate significant differences at *P* ≤ 0.05 with Tukey’s multiple comparison (ANOVA).

Numbers and body size of female offspring regressed with foundresses size, showing that the number of female offspring had a significant linear correlation with the body size of foundresses (*F* = 4.75, *df* = 1, 76, *P* = 0.032), but not with body size of their daughters (*F* = 0.006, *df* = 1, 76, *P* = 0.94). Large female wasps produced more daughters (Fig 3A), and body sizes of adult offspring produced by small mothers were the same as those produced by larger mothers (Fig 3C). Offspring sex ratios (the percentage of female wasps) were all strongly female–biased, with no difference among the three foundress size classes (*F* = 2.03, *df* = 2, 75, *P* = 0.14) (Fig 3B).

The duration of the egg stage was not affected by foundress size (*F* = 0.41, *df* = 2, 77, *P* = 0.66), nor was that of the pupal stage (male: *F* = 0.33, *df* = 2, 75, *P* = 0.72; female: *F* = 2.92, *df* = 2, 75, *P* = 0.06) (Table 2), but the duration of the larval stage differed significantly between offspring of small and large females: offspring produced by small female wasps needed more time to complete larval stage than offspring produced by large females (*F* = 3.80, *df* = 2, 77, *P* = 0.03) (Table 2). The total developmental duration of neither males nor females were not affected by the differences between their larval stages (male: *F* = 0.77, *df* = 2, 75, *P* = 0.47; female: *F* = 0.87, *df* = 2, 75, *P* = 0.42). The apparent contradiction is explained by the large standard error at each stage.

**Table 2:** The developmental duration of offspring and generation time of *Sclerodermus pupariae* with various body sizes on *Thyestilla gebleri* larvae.

Foundress size	Egg stage	Larval stage	Pupal stage (male)	Pupal stage (female)	Developmental duration of offspring (male)	Developmental duration of offspring (female)	Generation time of male wasps	Generation time of female wasps
**Small**	2.44 ± 0.51 a	6.67 ± 1.73 a	13.96 ± 0.77 a	15.04 ± 0.66 a	22.56 ± 3.68 a	23.59 ± 3.80 a	37.60 ± 2.96 a	38.68 ± 3.01 a
**Medium**	2.34 ± 0.48 a	5.57 ± 1.26 b	13.88 ± 0.91 a	14.82 ± 0.87 a	21.76 ± 1.52 a	22.71 ± 1.95 a	35.60 ± 2.93 b	36.54 ± 2.94 b
**Large**	2.44 ± 0.51 a	6.06 ± 1.76 ab	13.72 ± 1.27 a	14.44 ± 0.86 a	22.22 ± 1.63 a	22.94 ± 1.35 a	34.78 ± 2.34 b	35.5 ± 2.28 b

Data in the table refer to mean ± SE. Different letters within a column indicate significant differences among treatments at *P* ≤ 0.05 with Tukey’s multiple comparison (ANOVA).

### Generation time of *Sclerodermus pupariae*

The time to complete development of one generation was approximately one month and differed significantly between treatments (Table 2). The generation time of small female wasps was significantly longer than that of large females (male: *F* = 6.06, *df* = 2, 75, *P* = 0.004; female: *F* = 7.39, *df* = 2, 75, *P* = 0.001).

## Discussion

As they are widely used to control small and medium long–horned beetle larvae, bethylid parasitoids mass–reared on substitute hosts are an important part of successfully biological control. In the field, parasitoids avoided produce superfluous or feeble offspring depending on host size and host quality. However, due to the limited space and suboptimal inoculation proportions during the process of artificial reproduction, the parasitic efficiency of idiobiont adults and the fitness of offspring would be directly affected. In our study, small female wasps (< 2.4 mm) were found when parasitoid–host (*T*. *gebleri* larvae weighted 200.0–240.0 mg) ratios were more than 4:1 [40]. An optimal inoculation proportion depends on host species, body size, stage, mobility, behavior, and nutritional quality of the substitute host [41]. In the studies of congeneric *S*. *guani*, when it was reared on *Tenebrio molitor* Linnaeus, *Ptilineurus marmoratus* Reitter and *Saperda populnea* (Linnaeus), the optimal parasitoid–host ratios were 2:1, 4:3 and 1:5, respectively [42–44]. Once beyond the optimal ratios, small body size of offspring may be produced since the limited host nutrition and excessive eggs.

Because the host represents a finite resource, the fitness of a female parasitoid is partly determined by which she finds and parasitizes hosts [7]. Some field studies have shown that larger females are more likely than their smaller conspecifics to find hosts [45, 46] and to parasitize better quality (bigger) hosts [47]. Their parasitic abilities are also defined in terms of their “resource–holding potentials” [48]. These potentials, such as “fighting ability,” are intrinsic to the individual and are likely to increase with body size. In our study, small females had a higher cost than large females when attacking and conquering floundering hosts with same body size, reflected in higher mortality rate (small: 10/41; large: 0/23), longer paralysis time (small: 2.4 d; large: 1.67 d), and a lower parasitic success rate (small: 26/41; large: 18/23). On the contrary, if they encounter bigger or more active hosts, large females have greater advantages to subdue them than do smaller females.

Generally, large female parasitoids have greater reproductive potential because of larger volume of the spermatheca than small female. In our results, the brood size produced by large female (mean: 58.2) was also significantly larger than small females (mean: 42.2). Because the duration of pre–oviposition periods of various body size females were no significant difference S2B Fig., showing their host–feeding time were similar. It showed that large females did not store more sperm in a single mating, but they stored more resources and energy for reproduction than smaller female individuals [49, 50]. Moreover, other studies suggested that maternal body size does not influence the amount of sperm stored, but may be positively correlated with efficient management of the supply and transport of sperm [51].

The influences of maternal size on their offsprings’ sex ratio are not paid enough attention and only a few findings point that larger females produce a larger proportion of daughters [52–54]. This result may be explained by an adaptive sex ratio adjustment theory [55], because larger mothers are more likely to have an opportunity to acquire large, high–quality hosts that are better suited for the production of daughters, which they can more successfully exploit hosts [53, 54]. But in our study, there were little differences among test host larvae in body size and quality, so we concluded that the sex ratio of *S*. *pupariae* did not match the adjustment theory. Taylor (1981) reported that natural selection would cause the sex ratio of the mother parasitoid’s offspring to be biased towards the sex that competes for a limited resource more weakly than the other sex [56]. We found that the offspring of *S*. *pupariae* had a typically female–biased sex ratio in coordination with the local mate competition (LMC) theory, which is one of the most frequently referenced theories about the sex allocation made by parasitoids [57–59]. In other words, among the offspring of female *S*. *pupariae*, sons, but not daughters, competed for mates giving rise to the female–biased sex ratio. However, the female–biased sex ratios in Sclerodermus were explained by LMC, when only one foundress produced offspring on one host, but the ratios were best explained by local resource enhancement (LRE), when multiple foundress mutually exploited hosts [60].

To date, body size is considered to be the most prominent fitness measure in predicting clutch size and progeny resources allocation and little attention is given on the effects of development time on parasitoid fitness [12]. Because the development of eggs and pupae were relatively ‘static’, there is significant relationship between adult body size and larval feeding history [61, 62]. When the more offspring on a host produced, the greater competition for limited and shared food resources would be, which caused the growth rates of larvae to accelerate [60]. Then the larvae might choose for rapid development at the cost of reduced adult size [24, 39]. And related research showed that the combination of availably developmental duration and competition intensity of parasitoid larvae determined the amount of carry–over of resources to the pupal and adults stages [7, 63]. In our study, we found that although numbers of offspring were positively correlated with adult body size, body sizes and the duration of the egg stage was not affected by foundress size, nor was that of the pupal stage, but the duration of the larval stage of small female wasps was longer than that of large females. The results suggested that there was close relationship between brood size and developmental rate of parasitoid larvae and the larvae of large females had a higher degree of resource carry–over than these of small females, keeping consistent with the research results of *Trichogramma nubilale* [64]. Otherwise, as a gregarious parasitoid, the developmental duration of offspring produced by various body size mother wasps had no significant differences (male: 22 d; female: 23 d), showing that there were significant synchronicity in the development of offspring [65], such as the synchronicity of emergence [66]. Males of *S*. *pupariae* chew opened the cocoons of their sisters to mate with them [67] and improving developmental synchronization ensured that offspring mated and spread to seek hosts in a short time. Finally, a larger female had higher care efficiency to ensure more progenies development synchronously, because when eggs and larvae fell off the host, a large mother wasp could more easily transfer them back to host body surface in time for feeding than a smaller mother can.

In *S*. *pupariae*, body sizes of female progenies were independent of their mothers, indicating that body size of the foundress was not passed on to her children, and was not a maternal effect. It had shown that parasitoids depended on their mother to assess host suitability [68] and to allocate sufficient resources to each egg [69, 70]. Optimal clutch size theory also predicted that females produced a clutch size maximized their fitness gain per egg laid [7, 71]. Furthermore, the nutrition of each host larva used in our study was adequate for the progenies of a single synovigenic parasitoid. So we suggested that the phenotypes of offspring were caused by trade–off between clutch size and body size.

Currently, inoculative and inundative releasing of mass–reared *S*. *pupariae* for biocontrol small and medium–sized beetle larvae are identified as two of the most important approaches in China. Our results indicated that parasitic efficiencies of *S*. *pupariae* could be influenced by body size of foundress via the paralysis time, generation time, fecundity and maternal care provided in larval stage of offspring, whereas body size and female proportion of progencies were not affected. So there is need to choose reasonable body size of female wasps, to optimally utilize mass rearing and to control target pests with the lowest mortality cost. Furthermore, daily fecundity of *S*. *pupariae* was not measured, so its oviposition dynamic was not captured in our study and the trade–off between egg production and reproduction. Finally, because all of our studies were performed under laboratory conditions, the abilities of parasitoids with various body size to forage hosts and to adapt new environments need to be further investigated and comparative studies with these under field conditions.

## Supporting Information

S1 Fig The reproduction time of *Sclerodermus pupariae* with various body sizes. (a) paralysis time, (b) pre–oviposition period, and (c) oviposition period.
(EPS)

S2 Fig Relationship between body size of female wasps and fecundity (number of offspring emerged). Effect of body size on fecundity (number of offspring emerged) is significant.
(EPS)

S3 Fig Offspring performance of *Sclerodermus pupariae* with mothers of various body sizes. (a) numbers of female offspring, (b) sex ratio (proportion of females), (c) body size of female offspring.
(EPS)

S1 File Raw Data. File contains all data used in this manuscript.
(XLSX)

S1 Table Costs to females of *Sclerodermus pupariae* of attacking *Thyestilla gebleri* larvae. Different letters indicate significant differences at *P* < 0.05 (Chi–square test).
(DOCX)

S2 Table The developmental duration of offspring and generation time of *Sclerodermus pupariae* with various body sizes on *Thyestilla gebleri* larvae. Data in the table refer to mean ± SE. Different letters within a column indicate significant differences among treatments at *P* ≤ 0.05 with Tukey’s Multiple Comparison (ANOVA).
(DOCX)
