# Supplementary material for: Relationships between Body Size and Parasitic Fitness and Offspring Performance of Sclerodermus pupariae Yang et Yao (Hymenoptera: Bethylidae)
Source: PLoS One. 2016 Jul 1;11(7):e0156831. doi: 10.1371/journal.pone.0156831 (PMC4930212; doi:10.1371/journal.pone.0156831)
Supplement: S1 Table — Different letters indicate significant differences at P < 0.05 (Chi–square test). (DOCX) [file pone.0156831.s005.docx]

|  | **Number** | **Proportion** | **Proportion** | **Proportion** |
| --- | --- | --- | --- | --- |
|  | **Replicates** | **Wasps dead** | **Wasps that laid eggs** | **Wasps whose offspring emerged** |
| **Small wasps** | 41 | 10/41 a | 28/31 a | 26/28 a |
| **Medium wasps** | 47 | 6/47 b | 38/41 a | 35/38 a |
| **Large wasps** | 23 | 0/23 c | 22/23 a | 18/22 a |
| **Chi–square** |  | 7.29 | 0.55 | 2.03 |
| ***P*** |  | 0.03 | 0.76 | 0.36 |
