# Supplementary material for: Relationships between Body Size and Parasitic Fitness and Offspring Performance of Sclerodermus pupariae Yang et Yao (Hymenoptera: Bethylidae)
Source: PLoS One. 2016 Jul 1;11(7):e0156831. doi: 10.1371/journal.pone.0156831 (PMC4930212; doi:10.1371/journal.pone.0156831)
Supplement: S2 Table — Data in the table refer to mean ± SE. Different letters within a column indicate significant differences among treatments at P ≤ 0.05 with Tukey’s Multiple Comparison (ANOVA). (DOCX) [file pone.0156831.s006.docx]

| **Foundress size** | **Egg stage** | **Larval stage** | **Pupal stage (male)** | **Pupal stage (female)** | **Developmental duration of offspring (male)** | **Developmental duration of offspring (female)** | **Generation time of male wasps** | **Generation time of female wasps** |
| --- | --- | --- | --- | --- | --- | --- | --- | --- |
| **Small** | 2.44 ± 0.51 a | 6.67 ± 1.73 a | 13.96 ± 0.77 a | 15.04 ± 0.66 a | 22.56 ± 3.68 a | 23.59 ± 3.80 a | 37.60 ± 2.96 a | 38.68 ± 3.01 a |
| **Medium** | 2.34 ± 0.48 a | 5.57 ± 1.26 b | 13.88 ± 0.91 a | 14.82 ± 0.87 a | 21.76 ± 1.52 a | 22.71 ± 1.95 a | 35.60 ± 2.93 b | 36.54 ± 2.94 b |
| **Large** | 2.44 ± 0.51 a | 6.06 ± 1.76 ab | 13.72 ± 1.27 a | 14.44 ± 0.86 a | 22.22 ± 1.63 a | 22.94 ± 1.35 a | 34.78 ± 2.34 b | 35.5 ± 2.28 b |
